# Double-PEGylated Cyclopeptidic Photosensitizer Prodrug Improves Drug Uptake from In Vitro to Hen’s Egg Chorioallantoic Membrane Model

**DOI:** 10.3390/molecules26206241

**Published:** 2021-10-15

**Authors:** Jordan Bouilloux, Martin Kiening, Sopie Yapi, Norbert Lange

**Affiliations:** Laboratory of Pharmaceutical Technology, Institute of Pharmaceutical Sciences of Western Switzerland, School of Pharmaceutical Sciences, University of Geneva, CH-1211 Genève, Switzerland; jordan.bouillouxmaluret@gmail.com (J.B.); Martin.Kiening@unige.ch (M.K.); Sopie.Yapi@etu.unige.ch (S.Y.)

**Keywords:** photodynamic therapy, cyclopeptidic produgs, drug delivery, proteases, PEGylation

## Abstract

Cyclopeptidic photosensitizer prodrugs (cPPPs) are compounds designed to specifically target overexpressed hydrolases such as serine proteases, resulting in their specific activation in close proximity to tumor cells. In this study, we explored a series of conjugates that can be selectively activated by the urokinase plasminogen activator (uPA). They differ from each other by their pheophorbide a (Pha) loading, their number of PEG chains and the eventual presence of black hole quenchers (BHQ3). The involvement of a peptidic linker between the drugs and the cyclopeptidic carrier allows specific cleavage by uPA. Restoration of the photophysical activity was observed in vitro on A549 lung and MCF7 breast cancer cells that exhibited an increase in red fluorescence emission up to 5.1-fold and 7.8-fold, respectively for uPA-cPPQ_2+2/5_. While these cPPP conjugates do not show dark toxicity, they revealed their phototoxic potential in both cell lines at 5 µM of Pha_eq_ and a blue light fluence of 12.7 J/cm^2^ that resulted in complete cell death with almost all conjugates. This suggests, in addition to the promising use for cancer diagnosis, a use as a PDT agent. Intravenous injection of tetrasubstituted conjugates in fertilized hen eggs bearing a lung cancer nodule (A549) showed that a double PEGylation was favorable for the selective accumulation of the unquenched Pha moieties in the tumor nodules. Indeed, the diPEGylated uPA-cPPP_4/5_^2^ induced a 5.2-fold increase in fluorescence, while the monoPEGylated uPA-cPPP_4/5_ or uPA-cPPQ_2+2/5_ led to a 0.4-fold increase only.

## 1. Introduction

Cancer is one of the leading causes of death worldwide with more than 9.9 million deaths reported in 2020 [1]. Depending on the cancer type, incidence and mortality are not necessarily linked [2], implying that detection and treatment have to be performed as early and effectively as possible. In that respect, photodynamic therapy (PDT) is now well-established as a noninvasive technique for the treatment of various cancers, based on the trinity of light-photosensitizer-oxygen [3,4,5]. The photosensitizer (PS) is a compound that after topical or systemic administration accumulates selectively in target tissues in a time-dependent manner. Thus, researchers developed PSs from the first to third generation, by improving the photophysical characteristics thanks to chemical modifications. Then, biological considerations were implemented to improve PS delivery. Among the plethoric offer of designed variations, only a few of them could succeed to overcome all obstacles before reaching final approval for clinical use. In this context, PSs can be used for diagnosis and/or treatment of cancerous lesions [6,7,8,9].

In our group, we focused on proteolytically triggered drug delivery systems named polymeric photosensitizer prodrugs [10,11,12,13,14,15,16]. First, a poly(L-lysine) backbone was side-modified with solubilizing moieties such as PEG. Then, multiple copies of the fluorescent pheophorbide a (Pha) were coupled through a peptidic linker onto the backbone. This high loading resulted in a quenching of the free Pha’s fluorescence. Dismantling of these structures by the protease-mediated digestion of the peptidic linker enabled retrieval of the photophysical properties, thus allowing specific detection and PDT of cancerous cells in vitro and in vivo. We then translated this system into a more defined one by using a cyclodecapeptide as the building block of the conjugate. This template has a constrained planar structure due to its amino acidic structure, creating two specific reactive domains that qualify for multiple side modifications [17,18,19]. The defined structure of the cyclodecapeptide allows precise targeting of the position and number of PS moieties to load, resulting in strictly shaped final prodrugs. We demonstrated that despite the lower number of loaded PSs in our cyclopeptidic photosensitizer prodrugs (cPPPs) when compared to the polymeric system, fluorescence and ^1^O_2_ production were still effectively quenched in their native state [20,21].

We designed our prodrugs to have them active only at close proximity to cancerous cells. In this way, we tethered the PSs moieties onto the cyclopeptidic carrier through a peptidic sequence that will serve as a substrate for overexpressed enzymes. More precisely, we targeted the urokinase-like plasminogen activator (uPA), an enzyme involved in tumor proliferation and metastasis. This serine protease is reported to be overexpressed in several types of cancers [22,23], among others in human lung carcinoma and human prostate cancer. Furthermore, we drove our research on these types of cancer because the PDT treatments approved for lung cancer, such as Photofrin^®^ (Porfimer Sodium), show limitations including but not limited to severe and prolonged photosensitization and low activation wavelength that restricts the tissue penetration depth. Foscan^®^ (PEGylated formulation of meta-tetra(hydroxyphenyl)chlorin) however is not significantly selective for carcinomas in situ in the lung [24]. These studies allow a good comparison of pharmacokinetics in the chorioallantoic membrane model [25].

Additionally, TOOKAD^®^, the only PDT-based treatment for prostate cancer, was approved by the European Medicines Agency in 2017 for use in the European Union. However, the U.S. Food and Drug Administration voted against its approval in 2020, thus hindering its commercialization in the Unites States. This formulation, based on a palladium-bacteriopheophorbide (WST09, WST11) [26,27,28,29,30], is aimed at treating prostate cancer by vasculature-targeted photodynamic therapy (VTP). In our case, we do not rely on vasculature targeting to starve the tumor mass, but on the further internalization of our activated prodrug in the tumor cells themselves. This higher specificity is also linked to the specific increase in fluorescence emission when the palladium-bacteriopheophorbide alone did not present a good contrast for fluorescence emission.

## 2. Materials and Methods

### 2.1. Chemicals

Dulbecco’s phosphate-buffered saline (DPBS) solution without calcium and magnesium, 0.25% trypsin-EDTA solution, Dulbecco’s modified eagle medium (DMEM) with high glucose and GlutaMAX^TM^, penicillin 10,000 U/mL and streptomycin 10,000 µg/mL solution were provided by Life Technologies Corporation (Paisley, UK). Fetal calf serum (FCS) was purchased from Eurobio (Courtaboeuf, France). Matrigel^TM^ was obtained from Corning (New York, NY, USA). Dimethyl sulfoxide (DMSO) and MTT (3-[4,5-dimethylthiazol-2-yl]-2,5-diphenyltetrazolium bromide) were purchased from Sigma-Aldrich (Buchs, Switzerland). uPA-cPPP_1/5_, uPA-d-cPPP_D4/5,_ uPA-cPPP_4/5,_ uPA-cPPP_4/5_^2^ and uPA-cPPQ_2+2/5_ conjugates were synthetized as described previously [20,21].

### 2.2. Statistics

Graphics and statistics were generated using GraphPad Prism version 9.1.2 for Windows (GraphPad Software, San Diego, CA, USA).

### 2.3. Methods

#### 2.3.1. Cell Culture

A549 cells (ATCC^®^ CCL-185^TM^, Manassas, Virginia, USA) were cultured in DMEM medium. MCF7 cells (ATCC^®^ HTB-22™) were cultured in MEM medium. Culture media were supplemented with 10% (*v*/*v*) FCS and 1% (*v*/*v*) penicillin-streptomycin solution.

Cell lines were cultivated in a humidified incubator at 37 °C with 5% CO_2_ and routinely maintained by serial passage into fresh medium.

To prepare tumor spheroids, A549 cells were trypsinized, centrifuged and resuspended in cold complete culture medium supplemented with 3% Matrigel^TM^ to reach a concentration of 50,000 cells/mL. A total of 200 µL of this suspension was seeded into each well of a polyHEMA-coated round-bottom 96-well plate, leading to 10,000 cells per well. The plate was then centrifuged at room temperature for 10 min at 1000 rcf to induce clustering of the cells, then incubated in a humidified incubator at 37 °C with 5% CO_2_. Culture medium was renewed every two days by replacing 100 µL in each well.

#### 2.3.2. In Vitro Activation in A549 and MCF7 Cells

A549 and MCF7 were seeded at 10,000 cells per well in 96-well plates (in 75 µL of complete medium per well) and allowed to grow over 24 h. A stock solution of each conjugate at [Pha]_eq_ = 4 mM in DMSO was used to prepare 1, 2 and 10 µM solutions (at 2×) by dilution in complete medium, and 75 µL of these solutions were added to the cells to reach [Pha]_eq_ of 0.5, 1 and 5 µM. Live control was treated with complete medium and dead control with 50% DMSO.

Fluorescence level was followed over 24 h with a Safire microplate reader (Tecan, Switzerland) preheated at 37 °C. The following conditions were applied for all measurements: initial orbital shaking (slow strength, 5 s); λ_exc_: 410 ± 5 nm; λ_em_: 670 ± 5 nm; gain: 150; 10 flashes per well; integration time: 40 µs. Fluorescence emission levels were calculated using the following Formula (1):

(1) $$F_{r}=\frac{F_{t}-F_{0}}{F_{0}}$$

where $F_{r}$ is the relative fluorescence emission level, F_t_ is the fluorescence intensity measured at time t and F_0_ is the fluorescence intensity measured immediately after treatment. Values are expressed as mean value ± standard deviation. Plates were returned to the incubator when not being measured.

#### 2.3.3. In Vitro Dark Toxicity and PDT in A549 and MCF7 Cells

Following the in vitro activation, cells were rinsed twice with 100 µL of DPBS, followed by addition of 100 µL of fresh complete medium. Cells were then either kept in the dark or irradiated with a PCI Biotech Lumisource illuminator equipped with 4 blue light tubes (Osram L 18/67, 450 nm, 7.4 mW/cm^2^) at a blue light fluence of 12.7 J/cm^2^. Higher fluences that we tested were noxious on their own. Cell viability was assessed 24 h post-irradiation with the MTT assay. Cells were washed twice with 100 µL of DPBS and 50 µL MTT (650 mg/L of fresh complete medium) was added to each well. After 4 h of incubation in a humidified incubator at 37 °C with 5% CO_2_, 100 µL of DMSO was added into each well, followed by an additional incubation period of 30 min. Absorbances were then measured with a Safire microplate reader preheated at 37 °C. The following conditions were applied for all the experiments: initial orbital shaking (normal strength, 5 min); λ_abs_: 570 nm; 10 flashes per well. The cell survival was calculated using the following formula (2):

(2) $${\%}_{\mathrm{alive}}=100\times \frac{A_{i}-A_{x}}{A_{✓}-A_{x}}$$

where %_alive_ is the percentage of cell survival, A_i_ is the absorbance of the tested condition, A_✓_ is the absorbance of the live control and A_x_ is the absorbance of the dead control. Values are expressed as mean value ± standard deviation.

#### 2.3.4. Pharmacokinetics and Biodistribution in the Hen CAM Model

Upon receipt on embryonic development day 1 (EDD1), fertilized hen eggs (Animalerie Universitaire, University of Geneva, Geneva, Switzerland) were kept in an incubator (MG 200, Savimat, Chauffry, France) at 37 °C with a relative humidity rate of 65% for the whole duration of the experiment. Automatic rotation of the eggs was set until EDD_4_. At this time point, a 3 mm hole was drilled into the eggshell at the narrow apex and covered with an adhesive tape to allow detachment of the chorioallantoic membrane (CAM). At EDD_8_, the hole was enlarged to 2 cm to give access to the CAM. For tumor implantation, the CAM was perforated with a 25-gauge needle, the spheroid placed on top and fixed with 10 µL of Matrigel^TM^ and eggs sealed with parafilm. At EDD_12_, 30 µL of the conjugates were dissolved in saline water at [Pha]_eq_ = 0.8 mM and injected into one major blood vessel of the CAM with a 33-gauge needle fitted to a 100 µL syringe (Hamilton, Reno, NV, USA). Autofluorescence of the tumor was measured before injection and fluorescence images of the tumors were acquired over 23 h at relevant time points.

#### 2.3.5. Instrumentation Setup for CAM Imaging

Fluorescence imaging of the tumor nodules was achieved by using a Retriga EX camera (QImaging, Burnaby, BC, Canada) fitted to an Eclipse E600 FN fluorescence microscope (Nikon, Tokyo, Japan) equipped with a CFI achromat objective of 4x magnification and 0.1 numerical aperture. Illumination was performed with an HBO 103 W/2 mercury short-arc lamp (OSRAM Lich AG, Munich, Germany). The microscope was equipped with a BV-2A fluorescence cube (Nikon, Tokyo, Japan) composed of a medium-width bandpass excitation filter (400–440 nm), a longpass filter collecting fluorescence above 470 nm and a 455 nm cut-on wavelength dichromatic mirror. An additional ET665lp longpass filter (Chroma Technology Corp., Rockingham, VT, USA) was added to collect fluorescence above 665 nm. Images were treated with OpenLab 3.1.5 software (Improvision, Perkin Elmer, Coventry, UK).

## 3. Results and Discussions

### 3.1. Prodrug Approach

**Figure 1:**
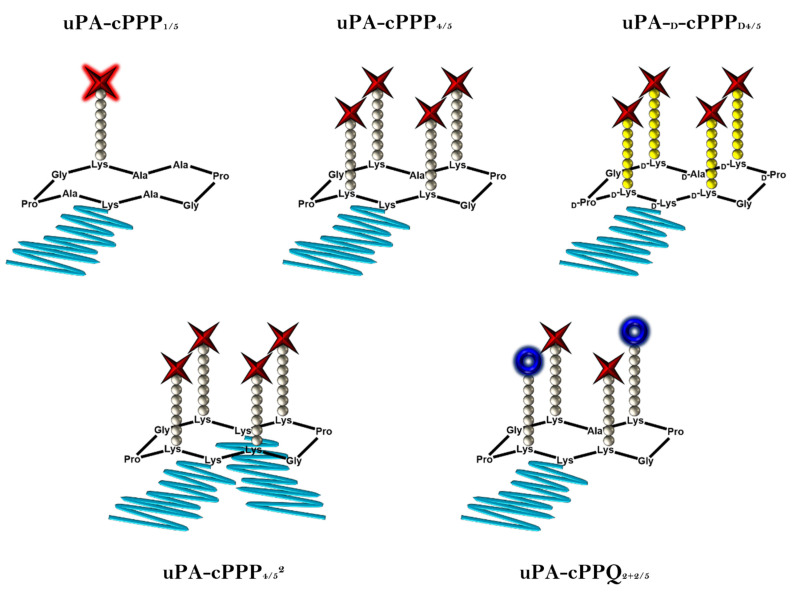
Schematic representations of the five prodrugs. Red stars stand for Pha moieties, blue crowns stand for BHQ3 moieties, grey and yellow balls stand, respectively, for l- or d-amino acids constituting the peptidic linkers and blue lines stand for PEG chains (5 kDa).

In previous studies [20,21], we demonstrated the rational use of a cyclopeptide-based template for the specific delivery of photosensitizers. This approach enables the elaboration of well-defined conjugates with accurate knowledge of the drug loading’s position and of the PEG side-chain modifications. Consequently, batch-to-batch variabilities are decreased and photophysical properties less dispersed. Here, the most promising conjugates have been investigated further in order to gain more information about their usability as prodrugs in photodynamic therapy. Additionally, a greater insight had to be gained regarding the biodistribution and pharmacokinetics at a more complex level. Figure 1 shows the five conjugates we tested, varying by the number and nature of drug loading and PEG side-chain modifications.

### 3.2. In Vitro Activation in A549 and MCF7 Cells

**Figure 2:**
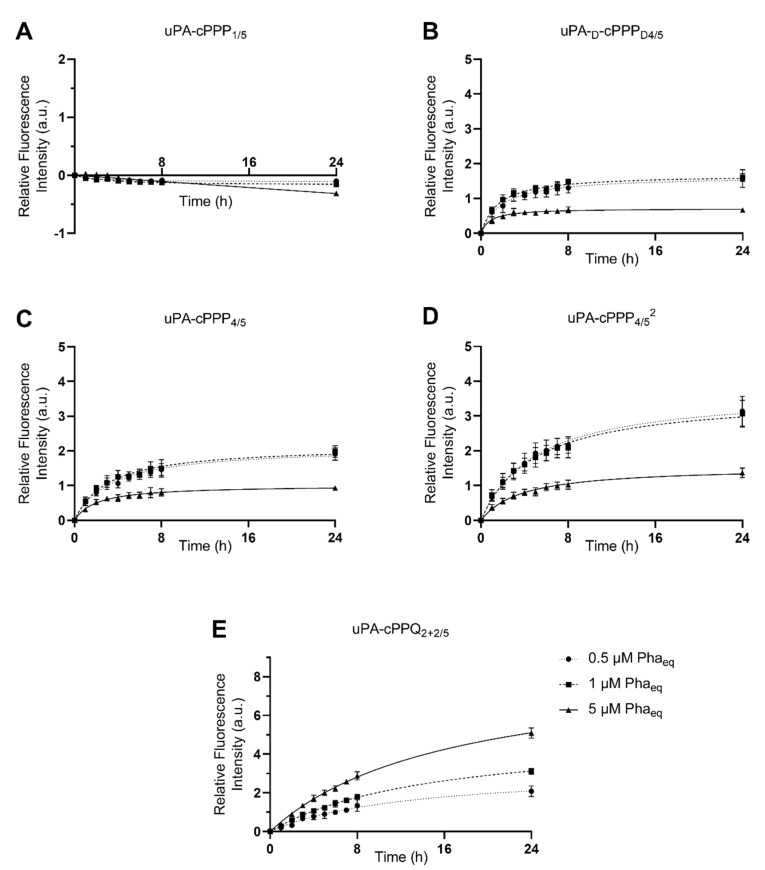
Red fluorescence emission of A549 cells upon incubation with 0.5 µM, 1 µM or 5 µM of (**A**) uPA-cPPP_1/5_, (**B**) uPA-d-cPPP_D4/5_, (**C**) uPA-cPPP_4/5_, (**D**) uPA-cPPP_4/5_^2^ or (**E**) uPA-cPPQ_2+2/5_ (n = 3 to 4; mean ± SD).

**Figure 3:**
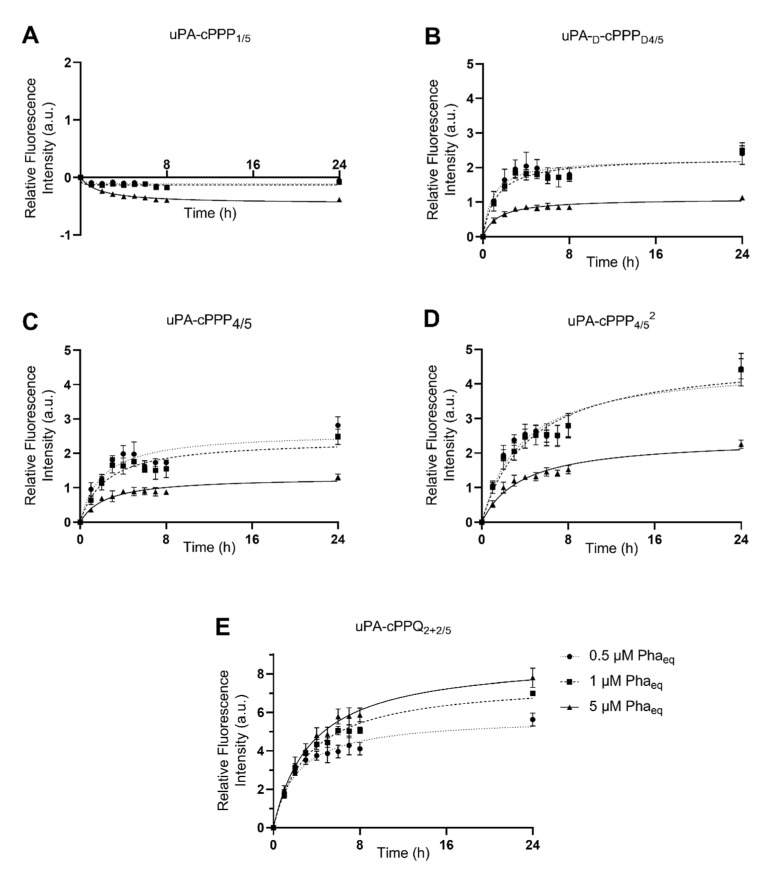
Red fluorescence emission of MCF7 cells upon incubation with 0.5 µM, 1 µM or 5 µM of (**A**) uPA-cPPP_1/5_, (**B**) uPA-d-cPPP_D4/5_, (**C**) uPA-cPPP_4/5_, (**D**) uPA-cPPP_4/5_^2^ or (**E**) uPA-cPPQ_2+2/5_ (n = 3 to 4; mean ± SD).

We assessed the ability of the five cPPP conjugates to generate in vitro red fluorescence emission in A549 and MCF7 cells, respectively originating from lung and breast adenocarcinomas. PDT is already approved for lung cancer [31] and A549 has been shown to produce high amounts of uPA [32]. The peptidyl linker used to tether the Pha and the BHQ3 moieties is constituted of the GSGRSAG sequence that acts as a substrate for uPA with the specific cleavage GSGR/SAG. We previously demonstrated that side-chain digestion occurs with serine proteases such as trypsin and uPA, resulting in the recovery of the fluorescence emission [11,16]. Here, we compare five conjugates that exhibit different behaviors. uPA-cPPP_1/5_ is Pha-monosubstituted and holds no quenching attribute in its initial state. On the other hand, uPA-cPPP_4/5_ and uPA-cPPQ_2+2/5_ are both highly quenched in their native states, the first one by short range interactions between the four Pha moieties and the latter by longer range interactions between the two Pha moieties coloaded with two BHQ3 moieties. In addition, we used uPA-d-cPPP_D4/5_ the analogous conjugate of uPA-cPPP_4/5_ whose usual L-amino acids were substituted with the equivalent d-amino acids to prevent its cleavage by uPA. Finally, the diPEGylated uPA-cPPP_4/5_^2^ was investigated for improved cellular bioavailability. Noticeably, the same behavior was observed when conjugates were incubated with cells or with recombinant enzymes. Unsurprisingly, uPA-cPPP_1/5_ (Figure 2A and Figure 3A) induced no significant increase of fluorescence emission over time in both cell lines, the digested Pha part that was internalized by cells being unquenched from the initial state. We could even notice a slight loss of fluorescence, probably due to the degradation or aggregation of Pha as suggested by the treatment with the highest concentration (5 µM) that results in higher fluorescence loss. However, uPA-d-cPPP_D4/5_ (Figure 2B and Figure 3B) treatment showed a release of Pha associated with an increase in the fluorescence level up to 1.6-fold in A549 and 2.5-fold in MCF7 at 24 h for 1 µM. While the uPA activity is targeted, it seems likely that another enzyme cut the GSGRSAG peptide, hence releasing the fluorescence emission. More investigations are required to obtain a comprehensive idea of which enzymes cut GSGRSAG in living cells. The L-analogous version uPA-cPPP_4/5_ (Figure 2C and Figure 3C) showed very similar profiles, albeit the answer was slightly higher in A549 (2-fold increase at 24 h for 1 µM). This difference may be due to the higher expression of the selective uPA or another relevant enzyme in A549 cells. The diPEGylation modification in uPA-cPPP_4/5_^2^ (Figure 2D and Figure 3D) enabled an increase in the relative fluorescence by 1.5 times in A549 and 1.8 times in MCF7 compared to the monoPEGylated uPA-cPPP_4/5_. Furthermore, we have recently demonstrated that a similar Doxorubicin diPEGylated construct (cPCP_4/5_^2^) was taken up by fibrosarcoma cells through late endosomes and lysosomes [33]. This supports the idea that PEG chains foster the bioavailability of the conjugate. Noticeably, all conjugates except uPA-cPPQ_2+2/5_ displayed 0.5 and 1 µM curves that superimposed, suggesting a saturation of uPA. Additionally, 5 µM of these conjugates produced a relative fluorescence by more than two times lower than 0.5 or 1 µM treatments. This reversal could be explained by the aggregation of the conjugates at high concentrations. In contrast, the introduction of BHQ3 moieties in uPA-cPPQ_2+2/5_ (Figure 2E and Figure 3E) produced the highest relative fluorescence intensity that increased with the concentration. After 24 h of treatment at 5 µM, the relative fluorescence was increased by 5.1 times in A549 and 7.8 times in MCF7. This phenomenon is presumably due to the very high initial quenching of the conjugate in its native state. Nonetheless, the recovery of dose-dependent kinetics may be due to a change in the conjugate’s structure and molecular weight and thus in its stability. In vitro digestion of the conjugate seems to allow a sufficient distance between the digested Pha-peptidyl and BHQ3-peptidyl moieties, preventing FRET quenching to occur. One can note that with uPA-cPPP_4/5_ a plateau is reached between 6 and 8 h post-incubation, whereas in the case of uPA-cPPQ_2+2/5_ the fluorescence recovery is still not complete 24 h after treatment. This might be due to possible interactions between Pha- and BHQ3-peptidyl moieties once internalized. Enzymes may also need a longer time to digest this specific construct that displays higher steric hindrance. The molecule may also be more stable, which could explain the dose-dependent fluorescence response that only happens when BHQ3s are grafted onto the template. Finally, uPA-cPPP_4/5_^2^ produced a relative fluorescence intensity still lower than uPA-cPPQ_2+2/5_ but 1.5 times higher in A549 and 1.8 times higher in MCF7 (at 24 h for 1 µM) than its monoPEGylated equivalent.

### 3.3. In Vitro Dark Toxicity and PDT in A549 and MCF7 Cells

The intrinsic toxicity of the compounds and their ability to induce phototoxicity were assessed in A549 and MCF7 cells. For this purpose, we treated the cells with uPA-cPPP_1/5_, uPA-d-cPPP_D4/5,_ uPA-cPPP_4/5_, uPA-cPPP_4/5_^2^ or uPA-cPPQ_2+2/5_ at concentrations of 0.5, 1 or 5 µM of Pha_eq_. Cells were then kept in the dark or irradiated with a blue light fluence of 12.7 J/cm^2^.

**Figure 4:**
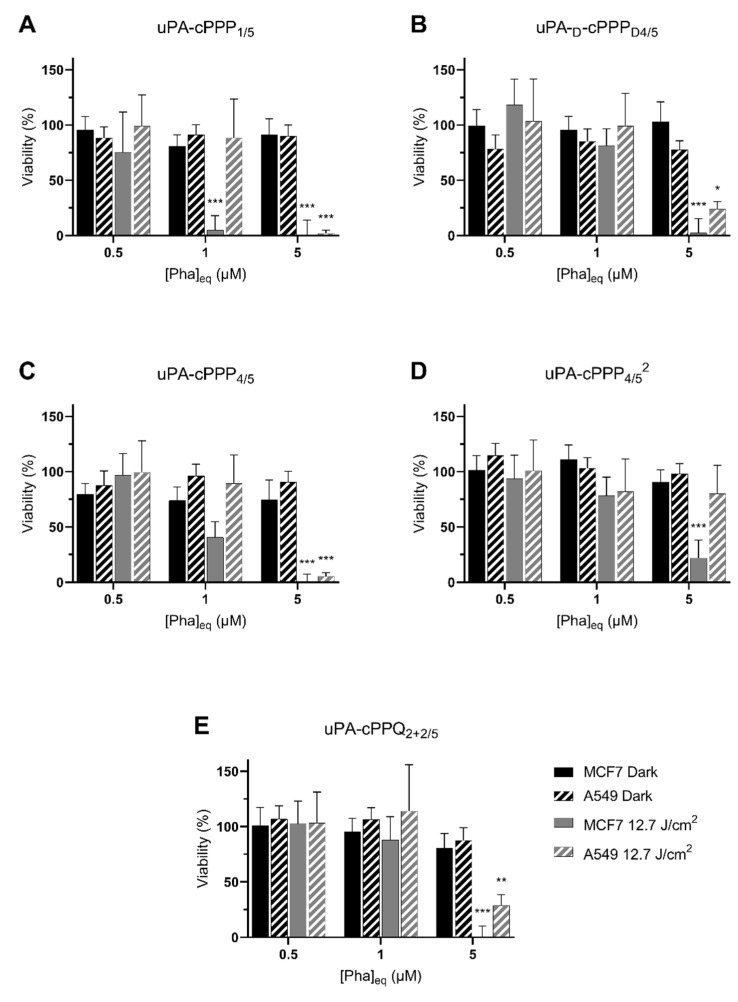
Phototoxicity at 24 h of 0.5 µM, 1 µM or 5 µM Pha_eq_ of (**A**) uPA-cPPP_1/5_, (**B**) uPA-d-cPPP_D4/5_, (**C**) uPA-cPPP_4/5_, (**D**) uPA-cPPP_4/5_^2^ or (**E**) uPA-cPPQ_2+2/5_ in A549 and MCF7 cells kept in the dark or irradiated with 12.7 J/cm^2^ of blue light (n = 3; mean ± SD; *: *p* < 0.05; **: *p* < 0.01; ***: *p* < 0.001; two-way ANOVA compared to the dark control at the same concentration of Pha equivalents).

None of the five conjugates showed dark toxicity. Even the highest concentration of 5 µM did not lower the viability of both cell lines (Figure 4). This supports the idea that the lower fluorescence that we observed after treatment with 5 µM of any conjugate except uPA-cPPQ_2+2/5_ was not due to a simultaneous cell death.

MCF7 cells were the most sensitive to PDT as they exhibited a 95% toxicity at 1 µM of uPA-cPPP_1/5_ (Figure 4A) and, although nonsignificant, a 60% toxicity at 1 µM of uPA-cPPP_4/5_ (Figure 4C). However, A549 showed no significant toxicity at 1 µM of any conjugate as the measured viability remained above 82% in all cases. At 5 µM though, the viability of MCF7 dropped close to 0% and the one of A549 under 30% with all conjugates except the diPEGylated uPA-cPPP_4/5_^2^ (Figure 4D) whose toxicity resulted in 22% and 80% viability, respectively. uPA-cPPQ_2+2/5_ (Figure 4E) did not show a PDT improvement. These results are quite promising since we are able to induce high toxicity with soft conditions. Comparatively, in a recent study by Song et al. [34], viability of HSC3 cells under PDT using the tirapazamine (TPZ)-loaded PEGylated chlorin e6 nanoparticles remained at 20% even under the harshest tested conditions (5 µg/mL of TPZ (~28 µM), 1 W/cm^2^ for 10 min (~600 J/cm^2^)). At conditions similar to ours (~12 J/cm²), Ruan et al. [35] used a BDP-PEGylated construct at 5 µg/mL of BDP (~7 µM) to be able to reach a 18% survival rate upon light treatment over HepG2 cancer cells, along with no dark toxicity.

Globally, we could hypothesize that at low concentration, the singlet oxygen production is too low to induce phototherapy or is quenched. uPA-cPPP_1/5_ is the sole conjugate not being quenched in terms of fluorescence but also singlet oxygen production. This may explain its induced higher phototoxicity that is unfortunately also present at the initial state in all tissues including the healthy ones. This lack of selectivity is however decreased in partially quenched tetrasubstituted conjugates and fully deleted when efficiently quenched with BHQ3.

It is important to notice that during the assay, cells were washed with DPBS prior to the irradiation step in order to remove any photoactive extracellular fragments. Consequently, and comparatively to the absence of photoactivity in the dark, only full constructs or at least big fragments that were internalized allowed singlet oxygen phototoxicity.

### 3.4. Pharmacokinetics and Biodistribution in the Hen CAM Model

Biodistribution and pharmacokinetic profiles are two essential points to ensure that PDT can be performed under optimal conditions, that is with the right prodrug concentration and at the right time post-treatment. The major part of active PS should be located in the tumor area to prevent any phototoxicity in the healthy surrounding tissues. That is why we moved from a basic 2D model using cancer cell lines to an advanced CAM 3D model, thus obtaining a better insight into the most promising tetrasubstituted conjugates. This is a crucial step before experimenting on an appropriate animal model in order to decrease the number of required animals and to optimize the treatment conditions (concentrations, time-windows, etc.) in the current 3R context required by the scientific community.

**Figure 5:**
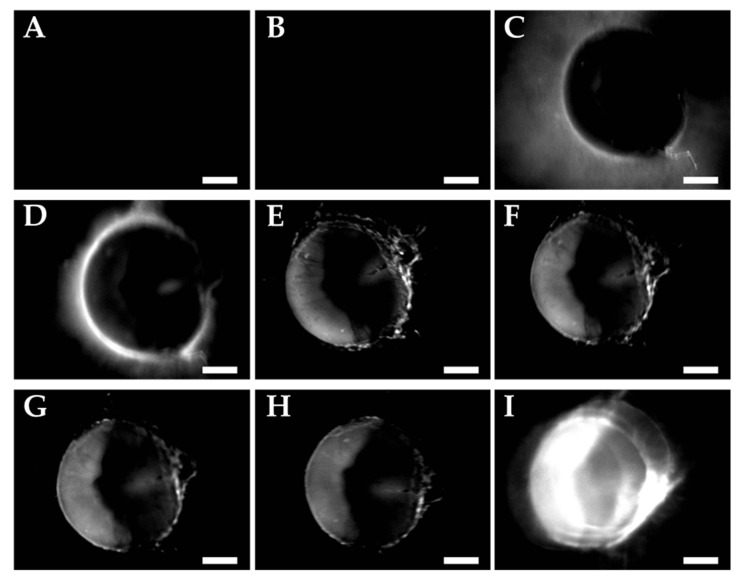
Fluorescence emission images of A549 nodules incubated with uPA-cPPP_4/5_^2^ (30 µL in NaCl 0.9% at 0.8 mM of Pha_eq_) (**A**) before injection, (**B**) 5 min, (**C**) 1 h, (**D**) 2 h, (**E**) 3 h, (**F**) 4 h, (**G**) 5 h, (**H**) 6 h and (**I**) 23 h after injection. Scale bar of 500 µm.

The activation of uPA-cPPP_4/5_^2^ is detected as a rise in fluorescence production over time inside the tumor and reaches its maximum at 24 h post-injection (Figure 5I). Knowing that uPA is excreted extracellularly, it is not surprising to notice a localization of the fluorescence outside of the tumor nodules at 1 and 2 h (Figure 5C,D). Thus, the two-hour period post-injection seems to express the adequate timeframe to enable the specific accumulation of the conjugate in the immediate vicinity of the tumor and its subsequent activation. Starting from three hours post-injection (Figure 5E), the fluorescence outside of the nodule is eliminated while it starts increasing inside cancerous cells. We could explain this evolution as the preferential uptake of the Pha-peptidyl fragment by the cancer cells rather than a clearing by the blood system. Similarly, the PEGylated PSSBDP construct developed by Ruan et al. [35] provided a higher uptake than the free BDP, demonstrating that PEGylation is of high importance when delivering small active molecules. By comparison, while in our case separation of the Pha from the PEGylated template occurred outside the cells upon proteolytic cleavage of the peptidic linker, their disulfide linker between PEG and BDP was cleaved upon GSH action only once internalized. Thus, a size limitation of the PEG moiety must be considered if one wants its construct (or at least its active part) to be internalized. In our case, we used one or two bigger PEG side chain(s), hoping that the resulting small active fragments would be rapidly internalized and not flushed through the bloodstream. Nevertheless, in a similar work [33] whose construct was based on a cathepsin B-sensitive peptidic linker, activation by this lysosomal protease was observed in the late endosomes and lysosomes, demonstrating that the full construct could be internalized into cells prior to being cleaved. If one wants to cleave the PEG sidechain before internalizing the drug into the cells, the linker might also be designed to react to ROS produced by a photosensitizer such as Ce6. In a study by Zhu et al. [36], the thioketal bond employed to PEGylate PLA nanoparticles was cleaved upon light irradiation after accumulation in the tumor tissue. This idea is quite interesting as one could not only cleave the PEG moieties in order to facilitate the internalization of the drug-loaded nanoparticles, but also initiate death of the cells through the production of ROS. The drawback of this method is that the user needs to be sure that the nanoparticles are accumulated specifically in the tumorous environment in order to avoid ROS generation in healthy tissue, since ROS would also be toxic for the surrounding cells. Our constructs do not display this problem since the generation of ROS will occur upon light activation only once the active Pha internalized.

The reliability of our constructs can be compared with those developed by Song et al. [34], where nontargeted CPT nanoparticles remained at the border of spheroids, whereas targeted CPTA nanoparticles were able to reach the core of tumor cell spheroids. The “targetability” of our conjugates, based on the cleavage of the peptidyl linker only at the vicinity of the tumor cells, appears to be relevant. We did not evaluate the depth of penetration of our active fragments, but we assume that it would be enough to trigger overall cellular death of the whole spheroid. This gives directions for further studies. In addition, one can notice that those nanoparticles seem to invade the tumor cell spheroid much faster than our conjugates: this might be due to the nanoparticle size, allowing them to accumulate faster, whereas our conjugates, as single molecules despite their 12–18 kDa range, need a longer time to accumulate.

**Figure 6:**
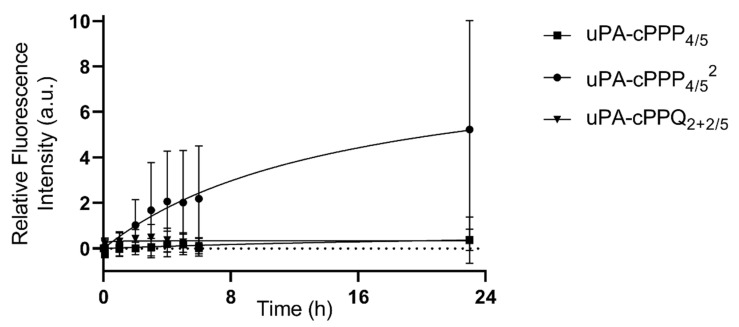
In vivo activation upon proteolytic digestion of uPA-cPPP_4/5_, uPA-cPPP_4/5_^2^ or uPA-cPPQ_2+2/5_ in A549 spheroids grafted onto hen egg CAMs (injection of 30 µL in NaCl 0.9% at 0.8 mM of Pha_eq_; n = 8 to 12; mean ± SD).

After 23 h of incubation, we observed a 5.2-fold relative increase in fluorescence emission for uPA-cPPP_4/5_^2^, when it was only 0.37-fold with uPA-cPPP_4/5_ and 0.38-fold for uPA-cPPQ_2+2/5_ (Figure 6). Only uPA-cPPP_4/5_^2^ is diPEGylated, which results in a higher molecular weight of ~18.0 kDa when compared with the monoPEGylated uPA-cPPP_4/5_ and uPA-cPPQ_2+2/5_ of ~12.5 kDa. The additional PEG chain is expected to increase the molecule’s solubility, but it may also change the 3D structure of the whole conjugate which could make it stay longer in blood circulation. Furthermore, the relative fluorescence intensity produced by uPA-cPPQ_2+2/5_ is always slightly higher than with uPA-cPPP_4/5_ between 0 and 8 h. This could be due to the presence of the supplementary cationic charges provided by the BHQ3 moieties that could interact with the cell membranes and slightly increase its residence time.

## 4. Conclusions

We developed well-defined cyclopeptidic photosensitizer prodrugs that are quenched in their native state at different levels. Their photophysical properties can be restored upon proteolytic digestion in the vicinity of tumors. A fluorescence emission increase was noticeable in vitro due to the specific cleavage of the peptidic linker between the template and the prodrug by the targeted urokinase plasminogen activator, serine protease. uPA has already been highlighted as a potent cancer prognosis tool as its level is often boosted in cancer cells due to its extracellular-matrix degradation feature. These prodrugs show no dark toxicity, but they induce cell death upon blue light irradiation. This toxicity depends on the drug concentration, which is correlated with a retrieval of ^1^O_2_ production by the photosensitizer-peptidyl fragment. The prodrugs are proteolytically activated outside of the tumor cells and the resulting fluorescent unquenched fragments are then internalized. A double PEGylation of the prodrug resulting in a bigger construct seems to be more adequate with regard to its pharmacokinetics and biodistribution. While we obtained a better activation in vitro with uPA-cPPQ_2+2/5_ than uPA-cPPP_4/5_^2^ in both cell lines, the latter performed outstandingly well in vivo.

These results bring forward the idea of creating an ultimate conjugate combining the two properties that enhanced the relative fluorescence production in cancer cells. Thus, we plan to design a diPEGylated conjugate bearing two Phas and two BHQ3s that would have the potential to overcome the current limitations.

## Sample Availability

Samples of the compounds are not available from the authors.
